# Runx2 activates PI3K/Akt signaling via mTORC2 regulation in invasive breast cancer cells

**DOI:** 10.1186/bcr3611

**Published:** 2014-01-30

**Authors:** Manish Tandon, Zujian Chen, Jitesh Pratap

**Affiliations:** 1 Department of Anatomy and Cell Biology, Rush University Medical Center, Armour Academic Center, 600 South Paulina Street, Suite 507, Chicago, IL 60612, USA

## Abstract

**Introduction:**

The Runt-related transcription factor Runx2 is critical for skeletal development but is also aberrantly expressed in breast cancers, and promotes cell growth and invasion. A de-regulated serine/threonine kinase Akt signaling pathway is implicated in mammary carcinogenesis and cell survival; however, the mechanisms underlying Runx2 role in survival of invasive breast cancer cells are still unclear.

**Methods:**

The phenotypic analysis of Runx2 function in cell survival was performed by gene silencing and flow cytometric analysis in highly invasive MDA-MB-231 and SUM-159-PT mammary epithelial cell lines. The expression analysis of Runx2 and pAkt (serine 473) proteins in metastatic breast cancer specimens was performed by immunohistochemistry. The mRNA and protein levels of kinases and phosphatases functional in Akt signaling were determined by real-time PCR and Western blotting, while DNA-protein interaction was studied by chromatin immunoprecipitation assays.

**Results:**

The high Runx2 levels in invasive mammary epithelial cell lines promoted cell survival in Akt phosphorylation (pAkt-serine 473) dependent manner. The analysis of kinases and phosphatases associated with pAkt regulation revealed that Runx2 promotes pAkt levels via mammalian target of rapamycin complex-2 (mTORC2). The recruitment of Runx2 on mTOR promoter coupled with Runx2-dependent expression of mTORC2 component Rictor defined Runx2 function in pAkt-mediated survival of invasive breast cancer cells.

**Conclusions:**

Our results identified a novel mechanism of Runx2 regulatory crosstalk in Akt signaling that could have important consequences in targeting invasive breast cancer-associated cell survival.

## Introduction

Breast cancer is the most commonly diagnosed form of cancer and a serious health concern for women worldwide [1]. One signaling mechanism that regulates breast cancer cell survival and is widely used to develop drug targets is the phosphatidyl inositol 3′ kinase (PI3K)-Akt pathway [2]. However, results from recent pre-clinical and clinical studies indicate a modest benefit from PI3K-Akt inhibitors as breast cancer cells acquire resistance due to feedback mechanisms and activation of other oncogenic signaling pathways [2,3]. Therefore, understanding the molecular basis of signaling crosstalk operative in cancer cells is required to improve the existing therapies and find novel strategies to control invasive breast cancers.

The Runt-related transcription factor, Runx2, is a key regulator of normal bone development, homeostasis and remodeling [4]; however, Runx2 is also aberrantly expressed in several cancer types, including breast [5,6], prostate [7], lung [8], ovarian [9] and osteosarcoma [10,11]. The Runx2 protein comprises structural motifs, including a DNA binding domain, nuclear localization signal (NLS) and nuclear matrix targeting signal (NMTS), for the localization of the protein into the nucleus [12]. The interaction of C-terminal domain of Runx2 with co-activators or co-repressors modulates downstream gene transcription in a context-dependent manner [13].

The invasive breast cancer-derived MDA-MB-231 cells express increased levels of Runx2 compared to non-tumorigenic MCF-10A cells [5]. The Runx2 overexpression in MCF-10A cells disrupts the acinar structures in three dimensional (3D) cultures and in poorly invasive MCF-7 cells induces epithelium to mesenchymal transition [14]. The Runx2 and its co-activator CBF-β regulates expression of matrix proteins and metalloproteinases (*MMP9* and *MMP13*), osteocalcin, bone sialoprotein and genes related to cancer cell migration and invasion [15-20]. These data highlight the invasive functions of Runx2 in breast cancers.

In response to epidermal growth factor (EGF) stimulation, the PI3K signaling pathway is activated, resulting in phosphorylation of serine/threonine kinase Akt (pAkt). The phosphorylation of Akt at Serine 473 residue is regulated by mammalian target of rapamycin complex-2 (mTORC2) and at Threonine 308 residue by phosphoinositide dependent kinase-1 (PDK1) [21,22]. The activity of mTORC2 complex depends on phosphorylation levels of mTOR at Serine 2448 and 2481 residues, and levels of Rictor and GβL proteins, while the mTORC1 complex includes Raptor instead of Rictor protein. The activation of Akt regulates downstream molecules (for example, MDM2, FOXO and GSK-3β) resulting in enhanced cell survival, proliferation and metabolism [23]. However, the effect of crosstalk of Runx2 and PI3K/Akt signaling for survival of breast cancer cells is still unknown.

In this study, we find that Runx2 suppression robustly enhances apoptotic cell death in invasive cancer cell lines in response to glucose- and growth factor-deprivation. We show that Runx2 is required for maintaining pAkt (Serine 473) levels in invasive mammary epithelial cell lines via mTORC2 complex proteins. Altogether, our results identify a novel mechanism implicating Runx2 regulatory network in the Akt cell survival pathway.

## Methods

### Cell culture, vectors and antibodies

All cell lines (*ATCC*, Manassas, VA, USA) were cultured at 37°C in humidified incubator with 5% CO2. For routine cell culture, the human MCF-10A cells (ER-, PR-, Her2+, WT-p53) [24] were cultured in Dulbecco's modified Eagle's medium (DMEM)/F12 media (*Cellgro*, *Mediatech*, Manassas, VA, USA) supplemented with 5% horse serum (*Lonza*, Walkersville, MD, USA), insulin (10 μg/ml) (USP Inc., Rockville, MD, USA), epidermal growth factor (EGF) (20 ng/ml) (*BD Biosciences*, Bedford, MA, USA), cholera toxin (100 ng/ml) (EMD Chemicals, Gibbstown, NJ, USA) and hydrocortisone (0.5 ug/ml) (*MP Biomedical*, Santa Ana, CA, USA), and 50 U/ml penicillin and 50 μg/ml streptomycin (pen-strep) (*Cellgro*, *Mediatech*). The human MCF-7 (ER+, PR + Her2+ WT-p53) [24] and MDA-MB-231 (ER-, PR-, Her2-, mutant-p53) [24] cells were cultured in MEM media (*Cellgro*, *Mediatech*) supplemented with 10% fetal bovine serum (FBS) (*Cellgro*, *Mediatech*) and pen-strep. The SUM-159 and SUM-159-PT (ER-, PR-, Her2-, mutant p53) [24] were cultured in F12 media (*Life Sciences Technology*, Grand Island, NY, USA) supplemented with 5% FBS, HEPES (10 mM), Insulin (5 μg/ml), hydrocortisone (1 μg/ml) and pen-strep. Since the cell lines used in this study were commercially obtained, the experiments performed in this study did not require approval from our institution’s ethics committee.

For EGF treatment, the cells were deprived of serum (final 0.25% serum) and growth factors, where appropriate, for 16 hours. The cells were then treated with EGF (100 ng/ml) in serum-deprived media for multiple time points (10 minutes to 6 hours). In experiments requiring PI3K inhibitor LY294002 (*Cayman Chemical,* Ann Arbor, MI, USA) treatment, the serum-deprived cells were pre-treated with LY294002 for 10 minutes before treatment with EGF or LY294002.

The mouse monoclonal antibody for Runx2 was obtained from MBL International Corporation, Woburn, MA, USA. The antibodies for pAkt (Serine 473 and Threonine 308), Akt (total), Akt1, Akt2, pPdk1 (Serine 241), pmTOR (Serine 2448 and 2481), mTOR (total), Rictor, Raptor, GβL, pGSK-3β (Serine 9) and FOXO1 were purchased from *Cell Signaling Technology*, Danvers, MA, USA*.* The antibodies for β-Actin and Lamin A/C were purchased from *Santa Cruz Biotechnology*, Santa Cruz, CA, USA.

### Transfection, transduction and stable cell line generation

The siRNA transfections for transient gene knockdown were carried out by using oligofectamine as per the manufacturer’s guidelines (*Invitrogen*, Grand Island, NY, USA). The following siRNA sequences were commercially (*Ambion*, Grand Island, NY, USA) synthesized: *PHLPP1*: (Sense) CCG UUG GAG UGA UGC ACA ATT. (Antisense) UUG UGC AUC ACU CCA ACG GCT; *Runx2*: (Sense) CUU GAU GAC UCU AAA CCU ATT. (Antisense) UAG GUU UAG AGU CAU CAA GCT.

The stable cell lines were generated utilizing lentivirus vectors. The lentivirus vectors expressing WT-Runx2, pLVTHM (RNAi-control) and Runx2-shRNA (Runx2-RNAi) were described and generated previously [5]. The lentiviral vector used to express constitutively active variant of Akt (CA-Akt) was kindly provided by Dr. Michael B. Johnson (Children’s Hospital of Philadelphia, PA, USA). The CA-Akt is deficient in pleckstrin homology domain (Δ4-129) and contains a Src myristoylation signal resulting in constitutive activation [25]. The early passage (<10) stable cells were utilized in the various assays. The adenovirus (Ad) vectors expressing green fluorescent protein (GFP) and WT-Runx2 were generated and described previously [26]. The lentivirus vector expressing *Rictor* shRNA was obtained from *Addgene* (plasmid #1853) (Cambridge, MA, USA) [27]. The doxycycline regulated knockdown of Runx2 was performed utilizing pLV-tTR-KRAB vector expressing the tetracycline repressor tTR-KRAB [28]. The tTR-KRAB binds to *tetO* operator in the absence of doxycycline to suppress shRNA, while in the presence of doxycycline it cannot bind to *tetO*, thus permitting the shRNA-mediated gene knockdown. The cells expressing pLVTHM (control or Runx2-shRNA) vectors harboring *tetO* were transduced with lentivirus expressing pLV-tTR-KRAB to generate doxycycline-induced Runx2 knockdown.

### Immunohistochemistry

The immunohistochemistry procedure was performed according to instructions in the Vectastain Elite ABC kit (*Vector Laboratories Inc*., Burlingame, CA, USA) but with some modifications. A human invasive carcinoma tissue microarray was obtained from *US Biomax, Inc*., (#BR1007) Rockville, MD, USA. This part of the study did not require approval from the Institutional Review Board as the tissue microarray was commercially sourced. Briefly*,* the standard histology procedures were used to deparaffinize the microarray slide in xylene and rehydrate it in graded ethanol series. The target retrieval was carried out by boiling the sections in citrate buffer (pH 6) (*Thermo Scientific*, Fremont, CA, USA) at 95°C for 15 minutes. The endogenous peroxidase activity was quenched by incubating slides in 3% hydrogen peroxide for 10 minutes. The blocking was performed with normal blocking serum (*Vector Laboratories*) for 30 minutes. The sections were subsequently incubated with control or anti-Runx2 or anti pAkt (Serine 473) antibody overnight at 4°C. The next day the sections were incubated in diluted biotinylated secondary antibody for 30 minutes at room temperature followed by 30-minute incubation with *Vectastain Elite ABC* reagent. The sections were thoroughly rinsed in PBS-T (PBS supplemented with 0.1% Tween-20) in between the above mentioned steps. The sections were finally incubated in peroxidase substrate solution to develop color, followed by washing in water, counter staining with hematoxylin (*Vector Laboratories*), clearing in xylene and cover-slipping with Permount (*Fisher Scientific*, Pittsburgh, PA, USA). The staining intensity for Runx2 and pAkt (Serine 473) were graded semi-quantitatively from 0 to 3 grades (0 = negative, 1 = low, 2 = medium and 3 = high) blindly by two investigators (MT and JP). The experiments were repeated three times.

### Western blotting

The whole cell lysates were prepared by washing cells in cold PBS and subsequently lysing in sample buffer containing Tris-Cl (62.5 mM, pH 6.8), SDS (2% w/v), DTT (50 mM), glycerol (10%) and bromophenol blue (0.01% w/v). The nuclear lysates were prepared in direct lysis buffer as previously described [5]. The whole cell and nuclear lysates were loaded in SDS-Gel and transferred to PVDF membrane and blotting was performed as previously described. The data were quantified in Adobe Photoshop (San Jose, CA, USA) and ImageJ software (NIH, Bathesda, MD, USA). All the experiments were repeated at least three times.

### Real-time PCR

The real time PCR with SYBR chemistry was performed as previously described [29]. The following human primer pairs were used. *Runx2*: (F) TGC CTG CCT GGG GTC TGT A (R) CGG GCC CTC CCT GAA CTC T; *mTOR*: (F) TCC GGC TGC TGT AGC TTA TTA (R) CGA GCA TAT GCC AAA GCA CT; *Rictor*: (F) CTT CGA GGA GGA CTA AAC AC (R) CTA CAT CAG CTC GCA CAT AC; *PHLPP1* (1): (F) CCT CAT CCG CTT CTA TGC AGG (R) GCATCTTGCCTTTACGGACAT; *PHLPP1* (2): (F) GCC AGT GAA CCG ATG GAC AA (R) GTC CCA CAT AGG ATG ACT TGG; *GAPDH*: (F) ATG TTC GTC ATG GGT GTG AA (R) TGT GGT CAT GAG TCC TTC CA; *28S*: (F) GAA CTT TGA AGG CCG AAG TG (R) ATC TGA ACC CGA CTC CCT TT. All the experiments were repeated at least three times.

### Chromatin Immunoprecipitation (ChIP)

The ChIP was performed as previously described [29]. The TF Search database was used to locate conserved Runx binding sites in *mTOR* promoter (−5,000 bases) [30]. The following primer pairs spanning Runx2 and *mTOR* promoter were used: *Runx2*: (F) GAA AGA GCA AGG GGG AAA AG (R) TGG AGA GGC AGA ATC ATG TG; *mTOR* promoter: (F) CAG TGG TGC AGT GGT GAG AT (R) AGG CAG GTG GAT TGT TTG AG. The experiment was repeated at least three times.

### Flow cytometry

The Annexin V and AAD staining was performed as per the manufacturer’s guidelines (*BD Biosciences*). The cell cycle analysis with propidium iodide staining was performed as previously described [31]. Briefly, the cells were harvested after trypsinization and fixed in ethanol for 24 hours in the cold. The fixed cells were washed in PBS supplemented with 2% FBS and suspended in PBS supplemented with RNAse (8 μg/ml) (*Sigma-Aldrich*, St. Louis, MO, USA) and stained with propidium iodide (18 μg/ml) (*Invitrogen*) for one hour. The cell cycle analysis was performed in FACS Canto (*BD Biosciences*). The gating and data analysis were performed in FlowJo software (*Tree Star Inc*., Ashland, OR, USA). The Dean Jett Fox model was used to set gates for G1, S and G2 stage cells, while Sub-G1 was manually gated before G1 population. The experiments were repeated three times.

### Cell proliferation

An indirect cell proliferation assay (*Promega*, Madison, WI, USA) was used to estimate cell number in triplicates at various time points in 96-well culture plates as previously described [29]. The media were supplemented with formazan dye and incubated for one hour. The absorbance was measured at 490 nm via spectrophotometer. The experiments were repeated three times.

## Results

### Runx2-depleted MDA-MB-231 and SUM-159-PT cells show increased apoptotic cell death with glucose- and serum-deprivation

**Figure 1:**
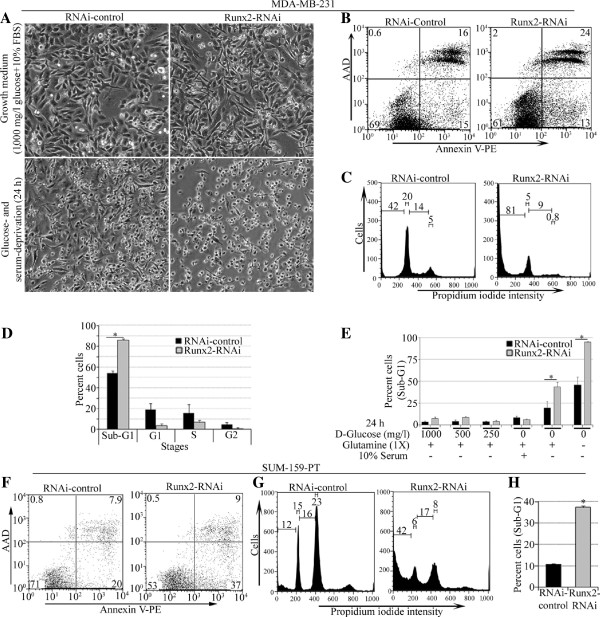
**Runx2 suppression increases apoptosis of invasive cells with glucose- and growth factor-deprivation. A)** The stable Runx2-RNAi or control MDA-MB-231 cells were deprived of glucose and serum for 24 hours. The morphology of these cells examined in brightfield microscope (10X) is shown. **B)** The Runx2 knockdown or control MDA-MB-231 cells were deprived of glucose and serum for 24 hours. The cells were then stained for Annexin V and AAD by flow cytometry. A representative cell population is shown. **C)** The glucose- and serum-deprived cells were examined for cell cycle by propidium iodide (PI) staining in flow cytometry. A histogram of representative cell cycle stages is shown. **D)** A quantification of average (± standard deviation) sub-G1, G1, S and G2 stages is indicated for PI stained Runx2-RNAi or control cells. **E)** The Runx2-RNAi or control MDA-MB-231 cells were placed in serum-, glutamine- and glucose-deprived medium or a medium substituted with glutamine and indicated doses of glucose for 24 hours. The fixed and PI-stained cells were then examined for sub-G1 population by flow cytometry. The percentage of sub-G1 cells in control or Runx2 knockdown cells is shown. **F)** The Runx2 knockdown SUM-159-PT or control cells were deprived of glucose and serum for 24 hours. The cells were then stained for Annexin V and AAD by flow cytometry. A representative cell population is shown. **G)** The glucose- and serum-deprived SUM-159-PT cells were collected and examined for cell cycle by PI staining in flow cytometry. A histogram of representative cell cycle stages is shown. **H)** A quantification of average (± standard deviation) sub-G1 stage is indicated for PI stained Runx2-RNAi or control cells. * indicates *P*-value <0.05 derived from unpaired Student’s *t*-test.

The invasive breast cancer cell lines and clinical specimens express high levels of Runx2 compared to non-tumorigenic breast epithelial MCF-10A cells (Additional file 1: Figures S1A, S1B, Additional file 2: Figure S2 and [5,6,15]). To determine the function of high endogenous Runx2, we suppressed Runx2 levels via lentiviral shRNA delivery in MDA-MB-231 cells (Additional file 1: Figure S1C) and performed cell proliferation and survival assays. The MDA-MB-231cells with Runx2 knockdown did not show any marked changes in cell proliferation compared to controls (Additional file 1: Figure S1E). Interestingly, when cultured in glucose- and serum-deprivation conditions, most pronounced changes were observed in Runx2 knockdown MDA-MB-231 cells. These cells became round and non-adherent within 24 hours compared to control cells (Figure 1A), suggesting increased cell death. The Runx2 knockdown cells revealed an increased (50% compared to control) Annexin V (a marker for early apoptosis) and AAD (marker for late apoptosis or dead cells) staining, indicating induction of apoptosis and loss of cell viability (Figure 1B). The transient Runx2 knockdown with a dsRNA targeting different regions in *Runx2* RNA also showed increased apoptotic cell death in response to glucose- and serum-deprivation (Additional file 1: Figure S1F). The cell cycle analysis of stable Runx2 knockdown cells revealed an over 35% increase in hypodiploid cells in Sub-G1 phase and a decline in G1 (from 19% to 3%), S (from 16% to 7%) and G2 (from 4% to 1%) phase compared to control (Figure 1C, D). The increase in Sub-G1 phase in Runx2 knockdown cells was partially restored by reconstituting the cell culture media with glutamine and was completely restored by reconstituting the media with 10% serum or 1,000 mg/l glucose (Figure 1E). We further validated the effect of Runx2 knockdown on cell death in another invasive breast cancer cell line, SUM-159-PT. The serum-, growth factor- and glucose-deprivation of SUM-159-PT cells with Runx2 knockdown (Additional file 1: Figure S1D) showed an increase in Annexin V staining (85% compared to control) for apoptosis (Figure 1F). The cell cycle analysis also revealed an over three-fold increase in Sub-G1 population (Figure 1G, H). These results suggest that Runx2 expression in invasive MDA-MB-231 and SUM-159-PT breast cancer cells protects from growth factor- and glucose starvation-induced cell death.

**Figure 2:**
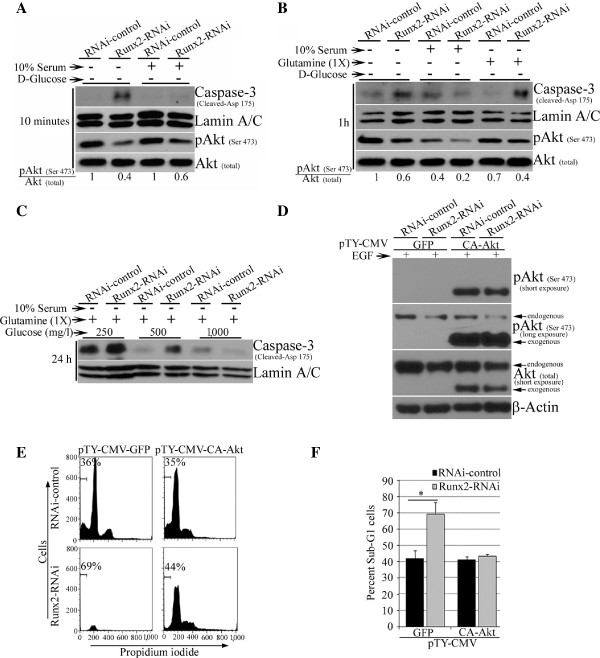
**Runx2 suppression increases caspase 3 cleavage in invasive MDA-MB-231 cells in response to glucose-deprivation. A, B, C)** The Runx2-RNAi or control MDA-MB-231 cells were deprived of glucose and serum for three hours. The cells were then left untreated or treated with 10% serum for 10 minutes **(A)**. The cells were then substituted with glutamine for 1 hour **(B)**, and indicated concentrations of glucose for 24 hours **(C)**. The whole cell lysates were examined for cleaved caspase-3, pAkt and total Akt expression, while Lamin A/C protein levels served as the loading control in Western blotting. **D)** The Runx2 knockdown or control MDA-MB-231 cells were utilized to re-express a constitutively active form of Akt (CA-Akt) or matching green fluorescent protein (GFP) vector control. The whole cell lysates were analyzed for Akt exogenous CA-Akt over-expression. **E)** The control or Runx2 knockdown MDA-MB-231 cells expressing CA-Akt or GFP were deprived of serum and glucose for 24 hours. The fixed cells were analyzed for sub-G1 population. **F)** The mean ± standard deviation of percent sub-G1 cells is shown. * indicates *P*-value <0.05 derived from unpaired Student’s *t*-test.

The Runx2 knockdown MDA-MB-231 cells with glucose- and serum- deprivation also showed an increase in caspase-3 cleavage, a hallmark of apoptosis, at multiple times (10 minutes to 24 h) compared to control cells as examined by Western blot analysis (Figure 2A-C) further confirmed the induction of apoptosis. The increased casapase-3 cleavage in Runx2 knockdown cells was rescued by reconstituting 10% serum, glutamine or glucose in the culture media (Figure 2B, C). Since Akt activity is essential for growth factor-induced cell survival, stimulation of glucose consumption in transformed cells [32] and high Runx2 expression associated with pAkt (Serine 473) positive specimens of invasive cancers (Additional file 2: Figure S2C-F), we examined pAkt (Serine 473) levels in Runx2 knockdown cells under serum- and glucose-deprivation. A corresponding decline in Akt phosphorylation (pAkt-Serine 473) was also observed in the Runx2 knockdown cells (Figure 2A, B). In order to investigate whether the effect of Runx2 depletion on cell survival in serum- and glucose-deprived conditions was mediated through pAkt, we over-expressed a constitutively active form of Akt (CA-Akt) in MDA-MB-231 cells. The exogenous expression of CA-Akt showed a robust increase in pAkt (Serine 473) levels (Figure 2D) and protected the Runx2 knockdown MDA-MB-231 cells (more than 25% compared to Runx2 knockdown alone) from serum- and glucose starvation-induced cell death (Figure 2E, F). Altogether, these results indicate that Runx2 activates Akt signaling and increases survival of invasive breast cancer cells in serum- and glucose starvation-induced cell death.

### Runx2 promotes Akt phosphorylation in highly invasive (MDA-MB-231) mammary epithelial cells

**Figure 3:**
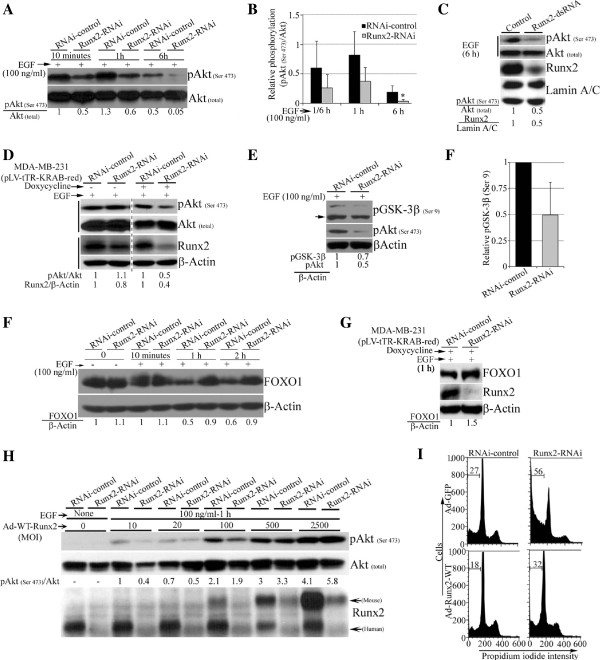
**Runx2 maintains pAkt levels in invasive MDA-MB-231 cells. A)** The MDA-MB-231 cells stably expressing Runx2-RNAi or control were serum-deprived and stimulated with epidermal growth factor (EGF) (100 ng/ml). The whole cell lysates were analyzed for pAkt (Serine 473) and total Akt expression by Western blotting at indicated times. **B)** The average (± standard deviation) of normalized relative pAkt levels post EGF stimulation at indicated time points are shown (*P <0.05). **C)** The MDA-MB-231 cells were transfected with dsRNA to transiently knockdown *Runx2* gene expression. The serum-deprived cells were stimulated with EGF and pAkt (Serine 473) level was analyzed. **D)** The *Runx2* gene expression was conditionally suppressed by doxycycline in tTR-KRAB expressing MDA-MB-231 cells. The serum-deprived cells were stimulated with EGF and pAkt (Serine 473) level was analyzed. **E)** The stable Runx2 knockdown MDA-MB-231 cells were examined for pAkt (Serine 473) and pGSK-3β (Serine 9) expression by Western blotting. **F)** The pGSK-3β (Serine 9) expression was quantified from three blots and normalized expression level is shown. **G** and **H)** The stable (G) or conditional (H) Runx2 knockdown MDA-MB-231 cells were stimulated with EGF at indicated times. The whole cell lysates were examined for the expression of FOXO1 protein, while β-Actin was used as loading control. **I)** The WT-Runx2 (mouse) was re-expressed by Ad vectors at indicated multiplicity of infection (MOI) in Runx2-RNAi MDA-MB-231 cells. The whole cell lysates of serum-deprived and EGF treated cells were analyzed for pAkt (Serine 473) and the total Akt protein expression by Western blotting. The re-expression of mouse WT-Runx2 was also confirmed. **J)** The control or Runx2 knockdown MDA-MB-231 cells were utilized to re-express WT-Runx2 (mouse) or green fluorescent protein (GFP) via Ad vectors and were deprived of serum and glucose for 24 hours. The fixed cells were analyzed for sub-G1 population. A representative cell cycle profile is shown.

In order to determine the function of Runx2 in the Akt signaling pathway, we examined levels of pAkt and its downstream genes in response to serum-deprivation and EGF stimulation in MDA-MB-231 cells. The serum-deprived control or Runx2 knockdown MDA-MB-231 cells when stimulated with EGF showed a potent induction of pAkt (Serine 473), a readout for the Akt signaling pathway activity (Additional file 3: Figure S3A, C). The Runx2 knockdown MDA-MB-231 cells showed reduction in pAkt at Serine 473 in response to EGF stimulation at multiple time points (10 minutes to 6 hours) examined (Figure 3A, B). A persistent decline in pAkt (Serine 473) was also observed with Runx2 knockdown with various doses (50 to 400 ng/ml) of EGF (Additional file 3: Figure S3A-D). We further confirmed the decline in pAkt levels by transient Runx2 knockdown using siRNA targeting a different Runx2 sequence (Figure 3C) and doxycycline-induced Runx2 knockdown (Figure 3D). To determine if Runx2 alters Akt activity, we assessed the phosphorylation of the downstream Akt target gene *GSK-3β*, and found that Runx2 suppression reduced the pGSK-3β (Serine 9) levels (Figure 3E, F). Since the phosphorylation of FOXO1 by pAkt promotes its degradation [33], therefore, we reasoned that the decline in pAkt levels associated with Runx2 suppression should result in increased FOXO1 levels. As expected, the Runx2 knockdown increased FOXO1 levels in the whole cell lysates stimulated with EGF (Figure 3G, H). The basal expression levels of Akt isoforms (Akt1 and Akt2), pAkt (Threonine 308) and pPDK1 (Serine 241) were only modestly affected in the Runx2-depleted MDA-MB-231 cells (Additional file 3: Figure S3E, F and data not shown).

To further establish the specificity of Runx2-mediated pAkt regulation, we re-expressed WT-Runx2 in Runx2-depleted MDA-MB-231 cells by Ad vectors. Although, an activating effect of Ad vectors on pAkt is known [34], the WT-Runx2 overexpression rescued the decline in pAkt (Serine 473), thereby establishing Runx2 function in maintaining pAkt levels (Figure 3I). The restoration of Runx2 expression was also sufficient to partially reduce the sub-G1 population observed in MDA-MB-231 cells in response to glucose- and serum-deprivation (Figure 3J). These results indicate that Runx2 is required for maintaining pAkt levels and survival of MDA-MB-231 cells.

### Runx2-mediated increase in Akt phosphorylation is specific for invasive cancer cells

**Figure 4:**
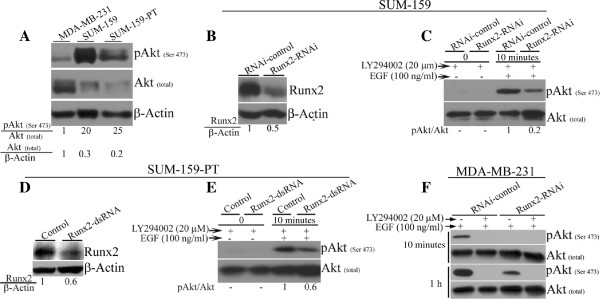
**Runx2 specifically maintains pAkt in invasive mammary epithelial cells. A)** The basal levels of pAkt (Serine 473) expression was examined in MDA-MB-231, SUM-159 and SUM-159-PT cells cultured in regular growth medium by Western blotting. **B)** The Runx2 protein expression was stably suppressed in SUM-159 cells by lentivirus-mediated Runx2 shRNA delivery. The Runx2 expression levels were analyzed by Western blotting while β-Actin was used as the loading control. **C)** The SUM-159 cells expressing Runx2-RNAi or control were serum-deprived and stimulated with 100 ng/ml of EGF in the presence of 20 μM LY294002. The whole cell lysates were analyzed for pAkt (Serine 473) and total Akt expression by Western blotting at 10 minutes post epidermal growth factor (EGF) stimulation. A quantification of normalized relative pAkt levels is given below respective lane. **D)** The SUM-159-PT cells were transfected with dsRNA targeting a different Runx2 mRNA sequence to transiently suppress Runx2 expression. **E)** The serum-deprived, EGF stimulated SUM-159-PT cells were analyzed for pAkt (Serine 473) and Akt levels by Western blotting. A quantification of normalized pAkt level is shown below the blot. **F)** The MDA-MB-231 cells expressing Runx2-RNAi or control were serum-deprived and stimulated with 100 ng/ml of EGF in the presence of 20 μM LY294002. The whole cell lysates were analyzed for pAkt (Serine 473) and total Akt expression by Western blotting at 10 minutes and 1 hour post EGF stimulation.

To determine whether decreased pAkt (Serine 473) levels with Runx2 suppression was specific for invasive cells, we examined additional invasive cell lines (Hs578t, HCC-38, SUM-159 and SUM-159-PT) with Runx2 knockdown and response to EGF treatment. Of these cell lines tested, SUM-159 and SUM-159-PT showed similar regulation as observed in MDA-MB-231 cells. As these cell lines have higher levels of endogenous pAkt (Serine 473) compared to MDA-MB-231 cells (Figure 4A), we utilized selective PI3K inhibitor, LY294002, to reduce basal pAkt levels. The Runx2 knockdown in SUM-159 and SUM-159-PT cells reduced pAkt (Serine 473) in EGF stimulated cells in the presence of LY294002 (Figure 4B-E). As expected, because of low levels of pAkt (Serine 473) in MDA-MB-231 cells, treatment with LY294002 resulted in complete abrogation of pAkt in both control and Runx2 knockdown cells (Figure 4F). These results indicate that endogenous Runx2 is required for maintaining pAkt levels in a subset of invasive breast cancer cells.

In non-invasive (MCF7) and normal (MCF-10A) cells, Runx2 knockdown (Additional file 4: Figure S4A, D) showed no change in pAkt (Serine 473) in the absence of LY294002 (Additional file 4: Figure S4B, E). Interestingly, in the presence of LY294002, increased pAkt (Serine 473) levels were detected (Additional file 4: Figure S4B, E). A quantification of average pAkt (Serine 473) expression levels upon EGF stimulation at multiple time points (one hour or less) in Runx2 knockdown MCF-10A and MCF-7 cells is shown in Additional file 4: Figure S4C, F. Taken together, these results show that Runx2-mediated activation of Akt signaling is specific for invasive mammary epithelial cells.

### Depleting PHLPP1 phosphatase and inhibiting ERK kinase activity restores pAkt levels in Runx2 knockdown MDA-MB-231 cells

**Figure 5:**
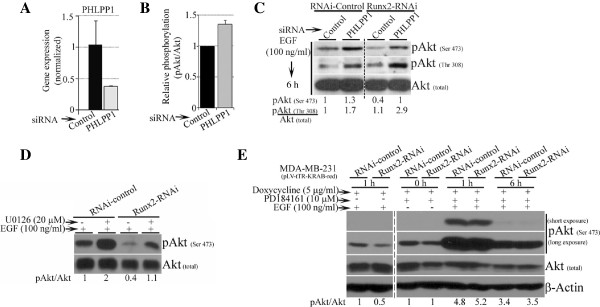
**Reducing PHLPP1 phosphatase and pERK kinase activity rescues Runx2-mediated inhibition of pAkt in MDA-MB-231 cells. A)** The *PHLPP1* mRNA expression was transiently suppressed in MDA-MB-231 cells and gene expression was analyzed by real-time PCR. A quantification of GAPDH normalized *PHLPP1* mRNA expression is shown. **B)** A quantification of the relative phosphorylation levels of pAkt (Serine 473) were determined by Western blotting in PHLPP1 knockdown MDA-MB-231 cells. **C)** The levels of pAkt (Serine 473) and total Akt proteins were analyzed by Western blot in PHLPP1 knock out control or Runx2 suppressed MDA-MB-231 cells as indicated. A quantification of pAkt levels normalized to total Akt protein is shown below the respective lane. **D)** The control or Runx2 knockdown MDA-MB-231 cells were stimulated with EGF for indicated times in the presence or absence of ERK inhibitor U0126. The level of pAkt and Akt was determined by Western blotting and quantification is shown below the respective lane. **E)** The *Runx2* gene expression was conditionally suppressed by doxycycline in MDA-MB-231 cells stably expressing tTR-KRAB and Runx2-shRNA. The serum-deprived cells were stimulated with EGF in the presence of ERK inhibitor PD184161 for indicated times. The pAkt and total Akt expression was analyzed by Western blotting and a quantification of normalized expression is shown below the respective lanes.

Several signaling events converge on the critical node of Akt for cell survival [35]. We investigated the effect of other regulators of pAkt on Runx2-mediated pAkt up-regulation. First, we examined PHLPP1 phosphatase that is known to dephosphorylate Akt (Serine 473) [36]. The knockdown of *PHLPP1* by siRNA in Runx2-depleted MDA-MB-231 cells partially restored pAkt (Serine 473) levels (Figure 5A-C). Secondly, a recent report indicated ERK-mediated inhibition of pAkt [37], therefore, we examined pAkt levels upon pERK inhibition in Runx2 knockdown cells. The inhibition of pERK by U0126 partially restored pAkt levels in Runx2 knockdown MDA-MB-231 cells (Figure 5D). To confirm this result, we used another specific pERK inhibitor, PD184161. The ERK inhibition with PD184161 showed a completely restored pAkt (Serine 473) levels in Runx2 knockdown cells (Figure 5E). The difference in the extent of rescue of pAkt (Serine 473) in Runx2 knockdown cells with ERK kinase inhibitors U0126 or PD184161 could be due to differences in the potencies of these two inhibitors. The PD184161 has been reported to be more potent in inhibiting phosphorylation of ERK1/2 than U0126 in human hepatocellular carcinoma cell lines [38]. These results suggest that, although Runx2 could function in PHLPP1 or ERK independent manner, Runx2-dependent pAkt up-regulation can be altered by inhibition of PHLPP1 or ERK activity.

### Runx2-mediated regulation of Akt phosphorylation is via mTORC2 complex proteins in invasive MDA-MB-231 cells

**Figure 6:**
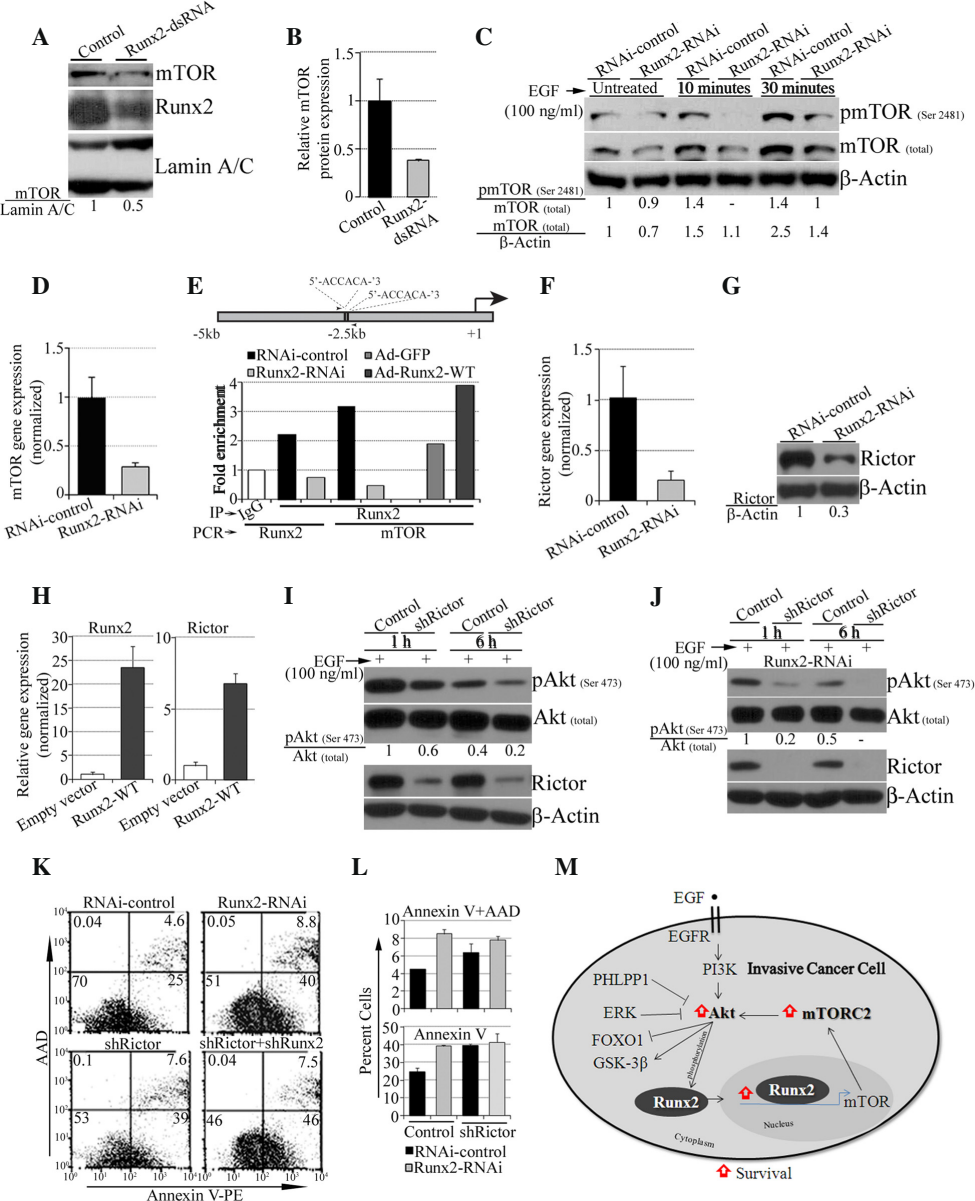
**Runx2 knockdown alters expression levels of mTORC-2 proteins. A)** The MDA-MB-231 cells’ transient Runx2 suppression was analyzed for mTOR and Runx2 levels. **B)** A quantification of mTOR protein expression normalized to β-Actin is shown. **C)** The stable Runx2 knockdown MDA-MB-231 cells were serum-deprived, epidermal growth factor (EGF) stimulated and examined for pmTOR (Serine 2481) and mTOR (total) protein. **D)** The Runx2 knockdown cells were assayed for *mTOR* gene expression by RT-PCR (normalized to *GAPDH*). **E)** The Runx2 knockdown and Ad-GFP- or WT-Runx2-treated MDA-MB-231 cells were tested for Runx2 recruitment on *mTOR* promoter by ChIP assays. A schematic diagram of the *mTOR* promoter region is bars indicating potential Runx binding sites (black bars) and location of PCR primers (small arrows). **F)** and **G)** The mRNA (F) and protein (G) expression levels of Rictor were analyzed in control or Runx2 knockdown MDA-MB-231 cells by RT-PCR and Western blotting, respectively. **H)** The MDA-MB-231 cells stably over-expressing WT-Runx2 were assayed for *Runx2* and *Rictor* gene expression by RT-PCR. **I)** The MDA-MB-231 cells with stable Rictor knockdown were stimulated with EGF and were analyzed for the expression of pAkt (Serine 473), Akt (total) and Rictor protein levels by Western blotting. **J)** The stably Runx2 knockdown MDA-MB-231 cells together with Rictor suppression were stimulated with EGF and analyzed for the expression of pAkt (Serine 473), Akt (total) and Rictor proteins by Western blotting. **K)** The glucose and serum-deprived (24 h) MDA-MB-231 cells with Runx2 knockdown (Runx2-RNAi) together with Rictor knockdown (Rictor -shRNA) were stained for Annexin V and AAD by flow cytometry. **L)** A quantification of positive cells with or without Rictor knockdown is shown. **M)** A schematic diagram depicting the function of Runx2 in regulating Akt via mTORC2 complex and its effect on survival of invasive cancer cells.

To understand the underlying molecular mechanism of Runx2-mediated up-regulation of pAkt in invasive MDA-MB-231 cells, we determined the expression levels of the mTORC2 subunit proteins, which are critical activators of Akt phosphorylation at Serine 473 [22,27]. The transient or inducible Runx2 knockdown in MDA-MB-231 cells reduced the expression level of mTOR protein (Figure 6A-C and Additional file 5: Figure S5A). The EGF treated Runx2 knockdown cells also showed reduction in phosphorylated mTOR protein at 10 and 30 minutes (Figure 6C) and no change at one hour (data not shown). The mRNA levels were also reduced more than two-fold in Runx2 knockdown cells compared to control cells (Figure 6D). In addition to MDA-MB-231 cells, Runx2 knockdown in SUM-159-PT cells also showed a decline in mTOR protein levels (Additional file 5: Figure S5B). To determine whether *mTOR* gene expression was directly regulated by Runx2, we examined binding of Runx2 on *mTOR* promoter region (5 kb upstream from transcriptional start site) by chromatin immunoprecipitation assays. Our result showed a direct binding of Runx2 on the *mTOR* promoter region (−2420 to −2441) containing two highly conserved Runx binding elements at −2420 and −2430 bp spanning 22 base pairs (Figure 6E). The ChIP assays revealed that endogenous and overexpressed Runx2 binds to *mTOR* promoter and that this binding is reduced by Runx2 knockdown. The *Runx2* promoter sequence was used as a positive control as previously is shown that Runx2 binds to its own promoter [39].

The expression of Rictor was also found to be reduced in Runx2 knockdown MDA-MB-231 cells and SUM-159-PT cell (Figure 6F, G and Additional file 5: Figure S5A, C, D). The WT-Runx2 over-expression in MDA-MB-231 cells increased Rictor expression, indicating that Rictor expression is Runx2-dependent (Figure 6H). The expression of the GβL subunit of the mTORC2 complex was minimally altered with Runx2 knockdown (data not shown). To further define the dependence of Runx2 on mTORC2 proteins in regulating pAkt and cell survival, we suppressed Rictor expression in parental or Runx2 knockdown MDA-MB-231 cells. The Rictor suppression reduced (40 to 50%) pAkt (Serine 473) levels compared to controls (Figure 6I), whereas the combination of Runx2 and Rictor knockdown reduced (80%) pAkt levels compared to Runx2 knockdown in response to EGF stimulation in MDA-MB-231 cells (Figure 6J). Furthermore, Rictor suppression alone or in combination with Runx2 robustly increased apoptotic cell death under glucose- and growth factor-deprivation compared to controls (Figure 6K, L). These results indicate that Runx2 activates the Akt signaling pathway and cell survival by regulating expression levels and activity of mTORC2 complex in invasive breast cancer cells.

## Discussion

The activated Akt signaling pathway, a common dysregulation in breast cancers, has been shown to promote cancer cell growth, survival and metastasis [2,3]. We find that Runx2 is required to maintain pAkt (Serine 473) levels in a subset of invasive breast cancer cells. Furthermore, we show that Runx2 regulates Akt survival signaling via mTOR and Rictor, key components of mTORC2 complex proteins. Interestingly, knockdown of Runx2 render these invasive cancer cells sensitive to apoptotic cell death in response to glucose- and serum-deprivation. Altogether, our findings indicate a novel Runx2 function in cell survival by regulating Akt signaling in invasive breast cancer cell lines (Figure 6M).

Previous studies have shown that functional mTORC2 complex is required for Akt phosphorylation at Serine 473 residue [27]. Consistent with this, our results show that down-regulation of pAkt (Serine 473) in Runx2 knockdown MDA-MB-231 cells is associated with a decrease in mTOR and Rictor protein levels of mTORC2 complex. Furthermore, the knockdown of Rictor in MDA-MB-231 cells reduced pAkt (Serine 473) levels and enhanced apoptotic cell death under glucose- and growth factor-deprivation. These results further support the dependence of functional mTORC2 complex on Runx2 in regulating pAkt levels. The binding of Runx2 on *mTOR* promoter could be influenced by recruitment of Runx2 regulatory complex (chromatin remodelers such as p300, CBP or HDACs) [19] or posttranslational modifications of Runx2 protein [40,41] further affecting basal expression levels and subsequent phosphorylation events of mTOR protein. We did not observe changes in expression levels of Raptor protein in MDA-MB-231 cells (data not shown) upon Runx2 modulation suggesting that mTORC1 is not involved in Runx2-mediated pAkt signaling.

The differential Akt regulation by Runx2 in non-invasive and invasive cancer cells could be due to altered Runx2 phosphorylation in invasive cells. Several reports indicate that in response to growth factor stimulation, phosphorylation and DNA binding activity of Runx2 is enhanced in osteogenic cell lines, endothelial cells and osteosarcoma cell lines [42-44]. In normal cell types, such as osteoblasts and chondrocytes, Runx2 DNA binding and Akt activity is shown to be interdependent during differentiation and cell migration of [42,45]. Interestingly, it has been reported that Runx2 is directly phosphorylated by Akt that increases Runx2 DNA binding activity in breast cancer cells (Figure 6M) [46]. Among other signaling events converging on the critical node of Akt [2,35], mutation of K-Ras (in MDA-MB-231 cells), PI3KCA (in SUM-159 cells) and p53 can also contribute to pAkt levels in invasive cell lines [24]. Higher endogenous levels of Runx2 have been reported in p53 null osteogenic and osteosarcoma cancer cells [47,48]. The down-regulation of p53 by Akt and inhibition of p53 transcriptional activity by the Runx2-HDAC complex have also been reported [2,35,47-50]. Based on these reports and our data in p53, mutant MDA-MB-231 and SUM-159 cell lines suggest a crosstalk among Runx2, Akt and p53 pathways [19,40-42,45]. High levels of Runx2 have been reported in breast cancers that correlated with clinical stage, histological grade and Her2 status in clinical breast cancer specimens [6]. Consistent with this report, our results show high levels of Runx2 and its association with pAkt (Serine 473), suggesting activation of Akt signaling in a subset of invasive cancers with high Runx2 expression. Our results in MDA-MB-231 cells indicate that Runx2 alters FOXO1 levels, a downstream effector of pAkt. Since FOXO1 has been shown to interact and inhibit the function of Runx2 in other cell types [51,52], it is likely that Runx2 directly interacts with FOXO1 protein as well as indirectly regulating its expression via modulating pAkt levels in mammary epithelial cells.

All three Runx transcription factors (Runx1, 2, 3) are shown to be expressed at varying levels in mouse and human normal or cancerous mammary epithelial cells [53,54] and alterations in the levels of these factors disrupt normal acinar structures of MCF-10A cells [5,55,56]. Consistent with the activation of PI3K/Akt signaling in MCF-10A cells [55], our findings also show a temporal regulation of EGF-induced pAkt levels by Runx2. Additionally, Runx1 and Runx3 proteins have been shown to regulate the PI3K/Akt pathway in megakaryocytic leukemic and gastric cancer cell lines by directly affecting expression levels of p110 and Akt1 proteins, respectively [57,58]. Collectively, these findings suggest that not only the relative levels of all three Runx proteins are important but these proteins may also regulate multiple effectors of the PI3K/Akt pathway.

Our studies in MDA-MB-231 and SUM-159-PT cells show that Runx2 knockdown increases cell death under glucose and growth factor-deprivation-induced stress. It has been shown in endothelial cells that glucose activates Runx2 phosphorylation, nuclear localization, DNA binding and cell migration [59,60]. These findings indicate that glucose metabolic signaling may synergize with Runx2 regulation of the Akt pathway, affecting post-translational modifications and enhancing downstream targets associated with cell survival. Taken together, our findings show that Runx2 promotes cancer cell survival by directly inducing subunits of mTORC2 kinase complex of the Akt signaling pathway and further suggest Runx2 inhibition as a potential therapeutic strategy in combination with currently available PI3K/Akt/mTOR inhibitors.

## Conclusions

We have demonstrated that Runx2 is required for phosphorylation of Akt via directly regulating sub-units of the mTORC2 complex. The Runx2-mediated pAkt regulation promotes survival of invasive mammary epithelial cells.

## Abbreviations

Ad: Adenovirus; Akt: Serine/threonine-specific protein kinase (PKB); CA: Constitutively active; CBFβ: Core binding protein β subunit; CBP: CREB-binding protein; ChIP: Chromatin immuno precipitation; EGF: Epidermal growth factor; ER: Estrogen receptor; ERK: Extracellular signal regulated kinase; FBS: Fetal bovine serum; FOXO1: Forkhead box protein o1; GFP: Green fluorescent protein; GβL: G-protein β subunit-like protein (mTOR associated protein); HDAC: Histone deacetylase; Her2: Human epidermal growth factor receptor-2; MMP: Matrix metalloproteinase; mTOR: Mammalian target of rapamycin; mTORC2: Mammalian target of rapamycin complex-2; MTT: Tetrazolium dye for colorimetric assay; NLS: Nuclear localization signal; NMTS: Nuclear matrix targeting signal; p300: E1A binding 300 kDA protein; p53: Protein 53; PBS: Phosphate-buffered saline; PCR: Polymerase chain reaction; PDK1: Phosphoinositide-dependent protein kinase-1; PHLPP1: PH domain leucine-rich repeat protein phosphatase-1; PI3K: Phosphatidyl inositol 3′ kinase; PR: Progesterone receptor; Runx2: Runt-related transcription factor-2; Ser: Serine; shRNA: Short hairpin RNA; siRNA: Small interfering RNA; Thr: Threonine; WT: Wild type.

## Competing interest

The authors declare that they have no competing interests.

## Authors’ contributions

All authors meet the authorship requirements. MT generated stable cell lines and analyzed cellular and molecular effects of Runx2 overexpression or knockdown (proliferation assay, ChIP assay, real time RT-PCR analysis, flow cytometry, Western blots). ZC performed real time RT-PCR analysis and Western blots of samples with Runx2 overexpression or knockdown. MT and JP participated in the data analysis. JP conceived and coordinated the study and MT and JP wrote the major part of the paper. All authors read and approved the final manuscript.

## Supplementary Material

Additional file 1: Figure S1 Runx2 expression levels in non-invasive or invasive cell lines and the effect of high Runx2 expression in MDA-MB-231 cells on proliferation and survival. Click here for file

Additional file 2: Figure S2 Relationship between Runx2 and pAkt (Serine 473) expression in invasive breast cancer specimens. Click here for file

Additional file 3: Figure S3 Runx2 knockdown reduces pAkt (Serine 473) when stimulated with various doses of EGF in MDA-MB-231 cells. Click here for file

Additional file 4: Figure S4 Runx2 knockdown increases pAkt (Serine 473) in non-invasive MCF-10A or MCF-7 cells in response to EGF stimulation in the presence of LY294002. Click here for file

Additional file 5: Figure S5 Runx2 knockdown in MDA-MB-231 or SUM-159PT cells alters expression levels of mTORC2 proteins. Click here for file
