# Supplementary figures and images for: Runx2 activates PI3K/Akt signaling via mTORC2 regulation in invasive breast cancer cells
Source: Breast Cancer Res. 2014 Jan 30;16(1):R16. doi: 10.1186/bcr3611 (PMC3979058; doi:10.1186/bcr3611)

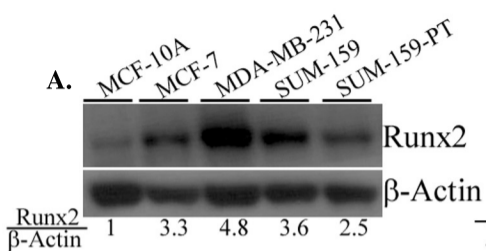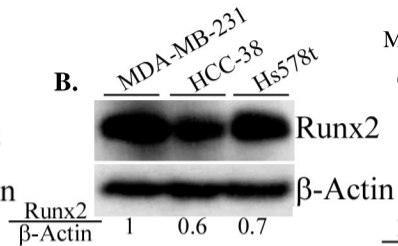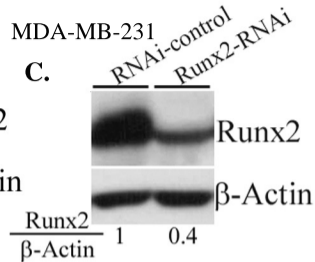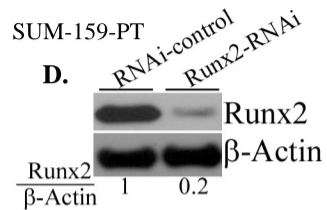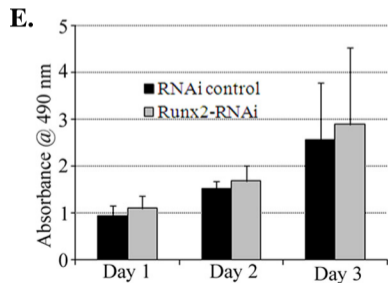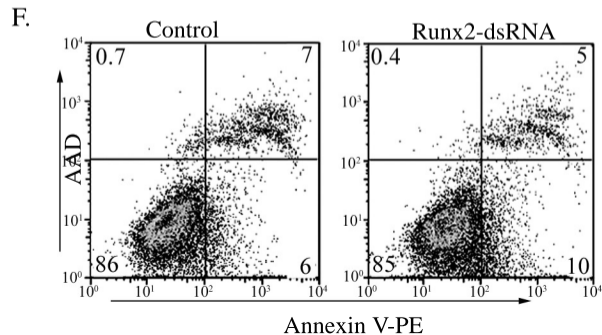

Supplement: Additional file 1: Figure S1 — Runx2 expression levels in non-invasive or invasive cell lines and the effect of high Runx2 expression in MDA-MB-231 cells on proliferation and survival. [file bcr3611-S1.pdf]

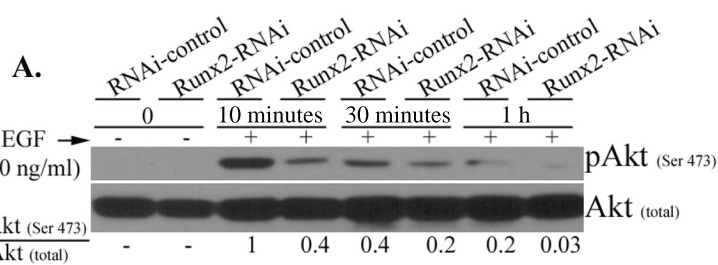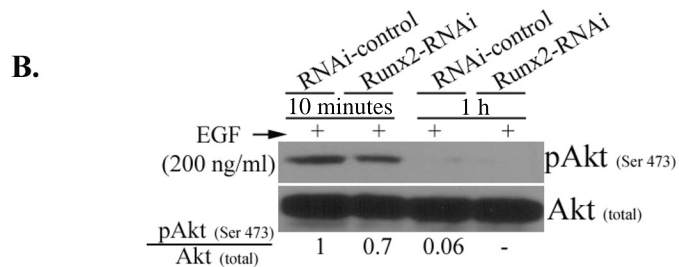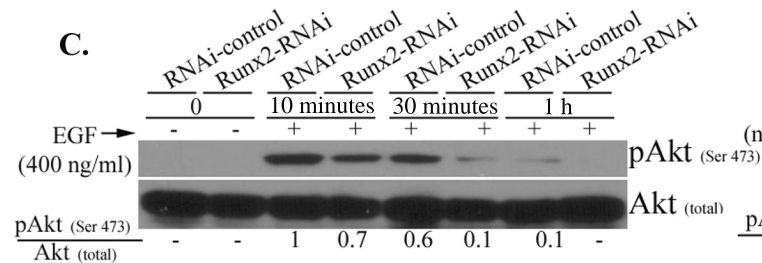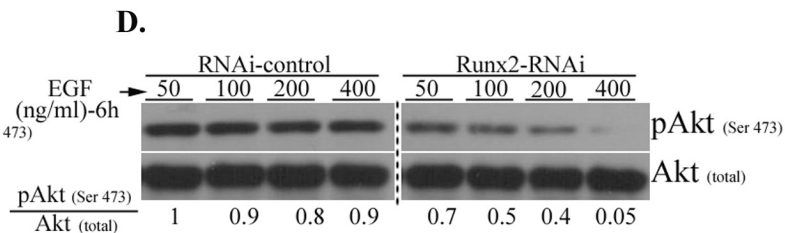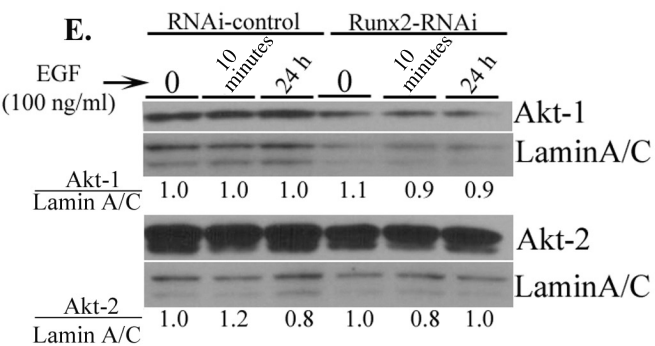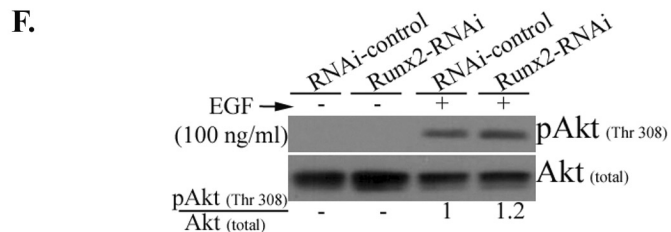

Supplement: Additional file 3: Figure S3 — Runx2 knockdown reduces pAkt (Serine 473) when stimulated with various doses of EGF in MDA-MB-231 cells. [file bcr3611-S3.pdf]

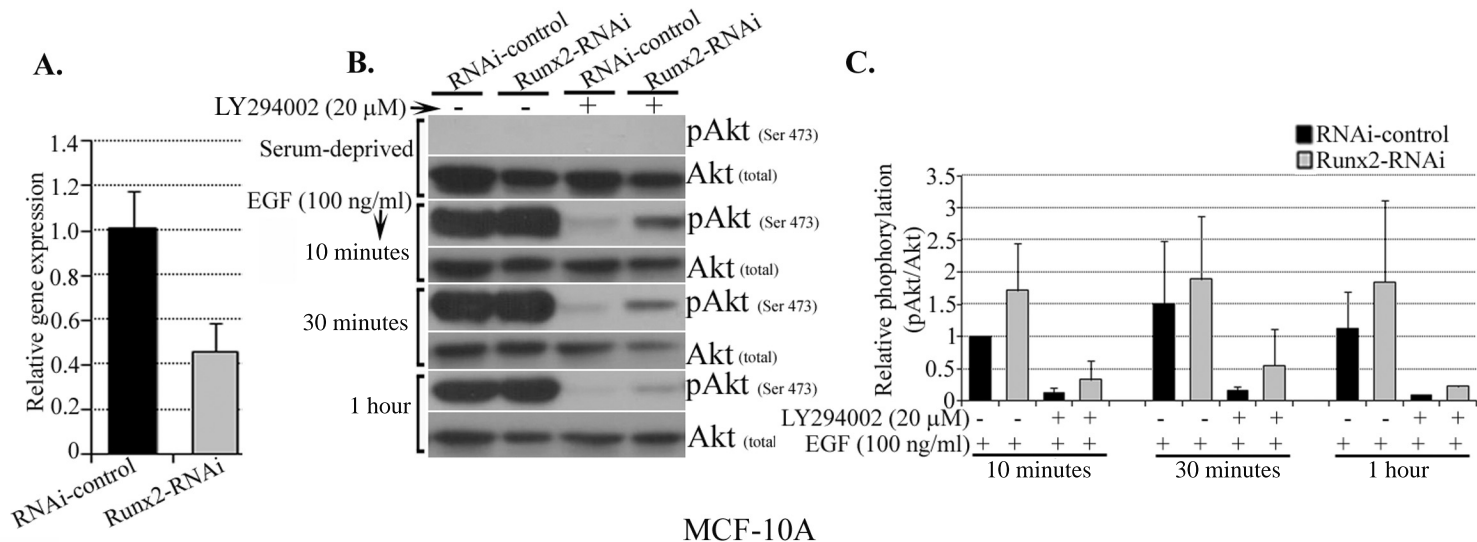

## MCF-10A

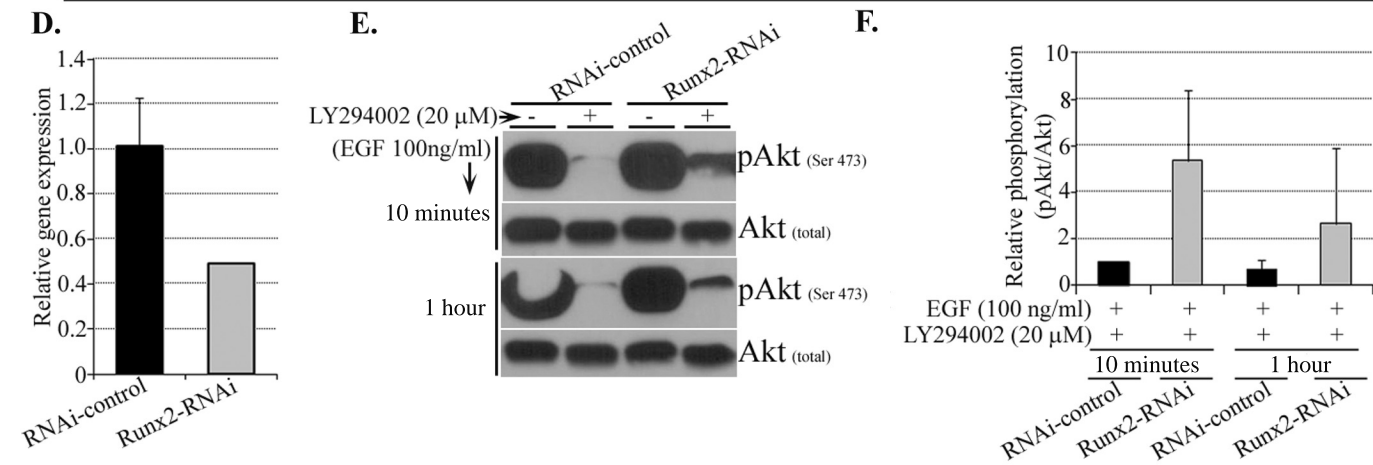

Supplement: Additional file 4: Figure S4 — Runx2 knockdown increases pAkt (Serine 473) in non-invasive MCF-10A or MCF-7 cells in response to EGF stimulation in the presence of LY294002. [file bcr3611-S4.pdf]

## MDA-MB-231

**A.** (pLV-tTR-KRAB)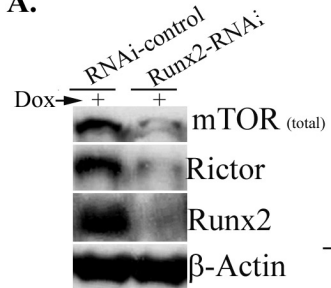

## SUM-159-PT

**B.**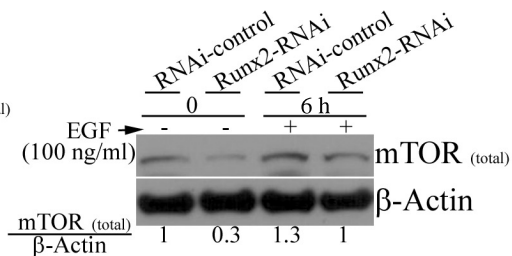**C.**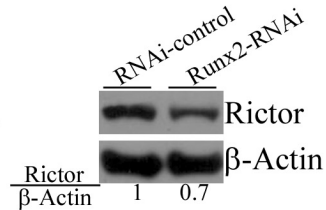**D.**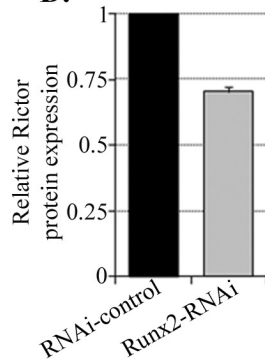

Supplement: Additional file 5: Figure S5 — Runx2 knockdown in MDA-MB-231 or SUM-159PT cells alters expression levels of mTORC2 proteins. [file bcr3611-S5.pdf]
