# Supplementary material for: Runx2 activates PI3K/Akt signaling via mTORC2 regulation in invasive breast cancer cells
Source: Breast Cancer Res. 2014 Jan 30;16(1):R16. doi: 10.1186/bcr3611 (PMC3979058; doi:10.1186/bcr3611)
Supplement: Additional file 2: Figure S2 — Relationship between Runx2 and pAkt (Serine 473) expression in invasive breast cancer specimens. [file bcr3611-S2.pdf]

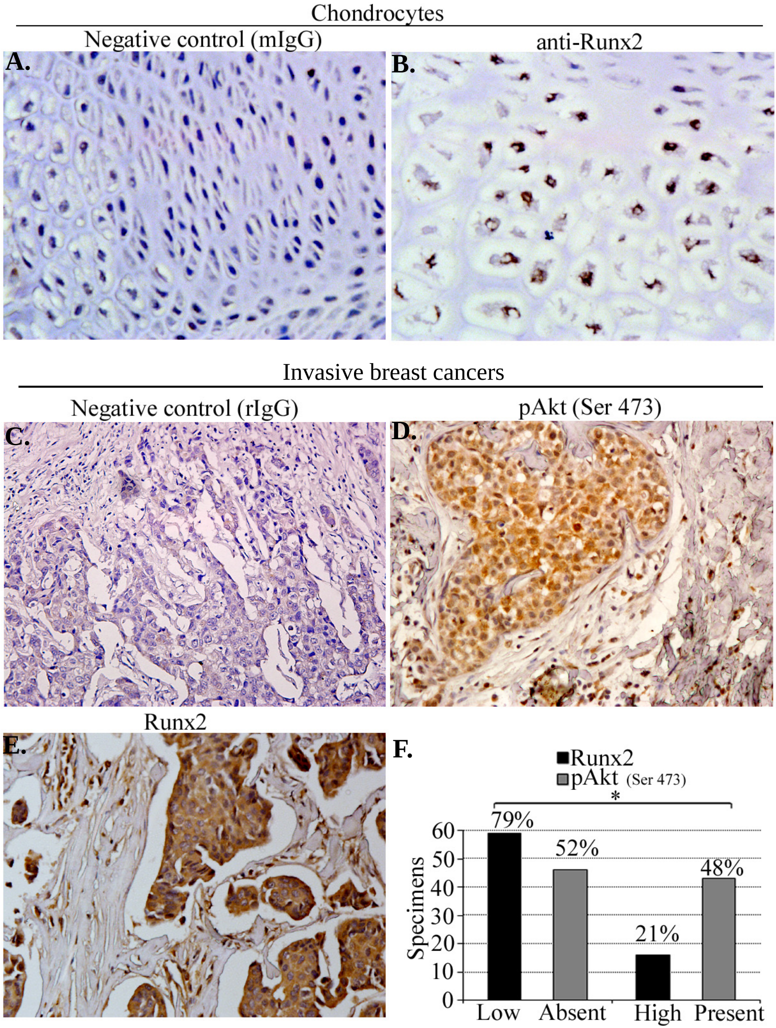
Chondrocytes
Negative control (mIgG)
anti-Runx2
A.
B.
Invasive breast cancers
Negative control (rIgG)
pAkt (Ser 473)
C.
D.
Runx2
E.
F.
Runx2
pAkt (Ser 473)
*
Specimens
0
10
20
30
40
50
60
79%
52%
21%
48%
Low
Absent
High
Present
